# Sanger Sequencing for *BRCA1* c.68_69del, *BRCA1* c.5266dup and *BRCA2* c.5946del Mutation Screen on Pap Smear Cytology Samples

**DOI:** 10.3390/ijms17020229

**Published:** 2016-02-08

**Authors:** Sin Hang Lee, Shaoxia Zhou, Tianjun Zhou, Guofan Hong

**Affiliations:** 1 Milford Hospital and Milford Molecular Diagnostics Laboratory, 2044 Bridgeport Avenue, Milford, CT 06460, USA; 2 State Key Laboratory of Molecular Biology, Institute of Biochemistry and Cell Biology, Shanghai Institutes for Biological Sciences, Chinese Academy of Sciences, 320 Yueyang Road, Shanghai 200031, China; sxzhou@njau.edu.cn (S.Z.); tjzhou@sibs.ac.cn (T.Z.); 3 College of Plant Protection, Nanjing Agricultural University, Nanjing 210095, China

**Keywords:** *BRCA1* c.68_69del, *BRCA1* c.5266dup, *BRCA2* c.5946del, Sanger sequencing, population screen, Pap smear cytology, HPV

## Abstract

Three sets of polymerase chain reaction (PCR) primers were designed for heminested PCR amplification of the target DNA fragments in the human genome which include the site of *BRCA1* c.68_69del, *BRCA1* c.5266dup and *BRCA2* c.5946del respectively, to prepare the templates for direct Sanger sequencing screen of these three founder mutations. With a robust PCR mixture, crude proteinase K digestate of the fixed cervicovaginal cells in the liquid-based Papanicolaou (Pap) cytology specimens can be used as the sample for target DNA amplification without pre-PCR DNA extraction, purification and quantitation. The post-PCR products can be used directly as the sequencing templates without further purification or quantitation. By simplifying the frontend procedures for template preparation, the cost for screening these three founder mutations can be reduced to about US $200 per test when performed in conjunction with human papillomavirus (HPV) assays now routinely ordered for cervical cancer prevention. With this projected price structure, selective patients in a high-risk population can be tested and each provided with a set of DNA sequencing electropherograms to document the absence or presence of these founder mutations in her genome to help assess inherited susceptibility to breast and ovarian cancer in this era of precision molecular personalized medicine.

## 1. Introduction

In the United States an estimated 231,840 new cases of invasive breast cancer are expected to be diagnosed in women and about 40,290 are expected to die from breast cancer in 2015 although death rates have been decreasing slightly since 1989 as the result of treatment advances, earlier detection through screening, and increased disease awareness [1]. For comparison, an estimated 21,290 women will be diagnosed with ovarian cancer and about 14,180 deaths from this disease will occur during the same period [2]. The high ratio of deaths over new cases among ovarian cancer patients indicates that ovarian cancer is even less treatable than breast cancer at the time of diagnosis. Since women with certain *BRCA1* and *BRCA2* germline mutations are at higher risk of developing breast cancer and ovarian cancer, identification of these harmful mutations can alert health care providers of the need for enhanced cancer surveillance in this subset of patients for early detection and the possibility of preventive interventions. Presymptomatic salpingo-oophorectomy and mastectomy for female *BRCA1* and *BRCA2* mutation carriers after child-bearing age can significantly reduce morbidity and mortality of these two cancers [3,4,5].

Modern genomics tools have uncovered thousands of missense mutations in the *BRCA1* and *BRCA2* genes; however, most of these mutations are of genetic variants of uncertain significance [6]. In theory, the newly developed next generation sequencing platforms can detect all these mutations. But to analyze their relevance as to the risk of developing breast cancer and ovarian cancer on every woman found to be a carrier of these mutations is challenging. As a result, several panels each consisting of a limited number of mutations have been proposed for selective screening [7,8,9,10]. These *BRCA1* and *BRCA2* mutation testing panels still cost up to US $4,000 per test in addition to the cost for genetic counseling, and population screening is not considered cost-effective in the U.S. unless the price can be lowered to US $250 per test, a threshold almost impossible to reach [11].

On the other hand, if testing for *BRCA1* and *BRCA2* focuses solely on unambiguously loss-of-function mutations with definitive effect on cancer risk [12], the cost per test may be markedly reduced. One study has found that of the 74 mutations identified in individuals with Ashkenazi ancestry through full sequence analysis of both *BRCA1* and *BRCA2*, only 16 (21.6%) were due to mutations other than *BRCA1* 1 c.68_69del(185delAG), *BRCA1* c.5266dup(5382insC), or *BRCA2* c.5946del(6174delT) [13]. Screening for these three founder mutations is sufficient to capture nearly all inherited cancer risk in this population due to *BRCA1* and *BRCA2* mutations [14]. The prevalence of these three mutations is approximately 10 times that of all *BRCA1* and *BRCA2* mutations in the general U.S. population [15]. Universal screening for these three founder mutations has been proposed for the women population in the Ashkenazi-Jewish community [14,16] as well as in other selective populations [12,17].

The purpose of this study was to develop a method for routine DNA sequencing-based testing for c.68_69del, c.5266dup and c.5946del mutations in conjunction with Pap smear and human papillomavirus (HPV) assays which are now performed for cervical cancer screening as part of the general gynecologic health care. Since one single proteinase K digestion of the fixed cervicovaginal cells can be used for all PCR amplifications, the additional cost for testing the three *BRCA* gene mutations may be as low as US $200. Since every laboratory report is accompanied by Sanger sequencing electropherograms to document the presence of a wild-type sequence or a sequence with mutation in these three target gene segments, the test results would be analytically self-validated. If these tests are implemented at the community hospital laboratory level, the practicing gynecologists may serve as the first-line health care providers to counsel every woman on her risk of developing cervical cancer, breast cancer and ovarian cancer with the help of these precision molecular diagnostics for better cancer prevention.

## 2. Results and Discussion

All nucleic acid-based molecular diagnostic tests are designed to determine the order of the four nucleotides within a gene of a pathogen or in the genome of a person, directly by DNA sequencing or indirectly by restriction fragment length polymorphism (RFLP), or by probe hybridization, including the different versions of PCR-based assays. When indirect methods are used for *BRCA* mutations testing, such as the multiplex-PCR test for 185delAG mutation [18], ambiguous results may be generated [19]. Several next-generation sequencing platforms have been developed in response to the call for high throughput sensitive precision *BRCA* screening [7,8,9,10]. However, Sanger sequencing remains the gold standard for nucleic acid-based tests, and cannot be totally replaced by next-generation sequencing, especially when the diagnostic signature sequence involves low-quality single-nucleotide variants and insertions or deletions <10 bp [20,21,22]. The choice of method for reliable population screening of the three founder mutations is unquestionably that by Sanger sequencing. The reason why Sanger sequencing has not been used in routine tests is the high cost associated with its implementation in clinical laboratory practice.

The major part of the cost in performing DNA sequencing-based tests on human materials is for sample preparation. In using the next generation sequencing technologies for *BRCA* mutation detections, the human genomic DNA needs to be isolated from EDTA blood by commercial extraction kits and spin columns. The isolated DNA must be checked for quality and quantified before being used for amplicon library construction by PCR [8,9,10]. For Sanger sequencing, human genomic DNA is also similarly isolated and quantified before being used for PCR amplification. Then the PCR products must be purified to remove the residual PCR primers before the amplicon can be used as the sequencing template [20,22]. In order to reduce the front-end cost for template preparation, we have chosen a robust low temperature heminested PCR system to enrich the target DNA fragments from crude proteinase K digestates of Pap smear cytology samples to be used as the templates for direct Sanger sequencing. Since no pre-PCR or post-PCR DNA purifications or quantitations are required, the cost for direct automated Sanger sequencing is markedly reduced.

### 2.1. LoTemp^®^ Nested PCR Amplicons as Sequencing Templates

Patient samples invariably contain inhibitors of primer-directed *in vitro* enzymatic DNA polymerization. Pre-PCR and post-PCR purifications by spin columns are usually required to remove theses inhibitors in all DNA sequencing-based diagnostic technologies. Even human genomic DNA itself may function as PCR inhibitors.

Our previous studies showed that crude proteinase K digestate of cervicovaginal cells fixed in alcohol-based liquid Pap cytology preservatives (ThinPrep^®^ or Surepath™) can be used directly to generate nested PCR amplicons which can serve as DNA sequencing templates for HPV genotyping without purification [23,24]. Since only 1 μL of the crude cell digestate is used to initiate a primary PCR in a 25 μL reaction volume, about 0.5 μL of the primary PCR products is transferred to a 25 μL nested PCR mixture by a micro-glass rod, and about 0.5 μL of the nested PCR products is transferred to a 20 μL Sanger reaction mixture, the polymerase inhibitors being carried over from the original crude proteinase K digestates are no longer an interfering factor in the Sanger reaction because all inhibitors in the digestate have been diluted ~50,000-fold (25 × 50 × 40). The major limiting factor in using nested PCR products for Sanger sequencing is the *Taq* errors and side-products accumulated in the nested PCR amplicons when the newly generated amplicons with *Taq* errors and mispriming are used as secondary templates during exponential replication of the target DNA copies if the total PCR thermal cycle number exceeds 40 when human genomic DNA is present [25].

In order to use nested PCR or heminested PCR amplification to prepare DNA sequencing templates while eliminating the need for pre-PCR and post-PCR purification steps, we have chosen a low temperature PCR system catalyzed by a moderately heat-resistant, high-fidelity DNA polymerase with high processivity [26] for DNA amplification. The clinical specimens used for the methodology development were 72 randomly selected archived, anonymized fixed human cervicovaginal cell suspensions, including 42 from women living in the USA and 30 living in Shanghai, China. These samples were preserved in commercial ThinPrep^®^ or Surepath™ fixatives and were the residues of the liquid-based Pap cytology specimens collected by local practicing gynecologists as part of the women’s health care protocols. The original samples were submitted for HPV assays, and these sample residues had been stored at various temperatures (−20 °C, 4 °C or ambient temperature) from a few weeks to 9 years before use for the present study. Publication of these laboratory data with blinded patient identities was approved by the Institutional Review Board of Milford Hospital [27] and by the Review Board of the Institute of Biochemistry and Cell Biology, Shanghai Institutes for Biological Sciences, Chinese Academy of Sciences. In addition, five samples of human cervicovaginal cells preserved in ThinPrep^®^ fixatives received from the New York State Department of Health, Clinical Laboratory Evaluation Program (CLEP) were also tested to confirm that the PCR primers selected are suitable for amplification of the target DNA fragments from different sources. Expanded testing on a larger volume of patient samples is ongoing to comply with regulatory requirements before this technology is offered as a clinical test to the public.

The three PCR primer sets chosen for preparation of the amplicons used as direct DNA sequencing templates were oligonucleotides with following sequences:

Heminested PCR primers for *BRCA1* c.68_69del detection

Primary PCR

Forward 5′-GAAGTTGTCATTTTATAAACCTTT-3′ [18]

Reverse 5′-GTATGTAAGGTCAATTCTGTTC-3′ [18]

Heminested PCR

Forward 5′-TCATTGGAACAGAAAGAAATGG-3′ (also as routine sequencing primer)

Reverse 5′-GTATGTAAGGTCAATTCTGTTC-3′

Heminested PCR primers for *BRCA1* c.5266dup detection

Primary PCR

Forward 5′-GTCTGCTCCACTTCCATTGAAG-3′

Reverse 5′-GATGGAAGAGTGAAAAAAGAAC-3′

Heminested PCR

Forward 5′-GAAGCTTCTCTTTCTCTTATCC-3′ (also as routine sequencing primer)

Reverse 5′-GATGGAAGAGTGAAAAAAGAAC-3′

Heminested PCR primers for *BRCA2* c.5946del detection

Primary PCR

Forward 5′-TTTGCTGACATTCAGAGTGAAG-3′

Reverse 5′-CTCTTGTGAGCTGGTCTGAATG-3′

Heminested PCR

Forward 5′-TCACCTTGTGATGTTAGTTTGG-3′ (also as routine sequencing primer)

Reverse 5′-CTCTTGTGAGCTGGTCTGAATG-3′

**Figure 1:**
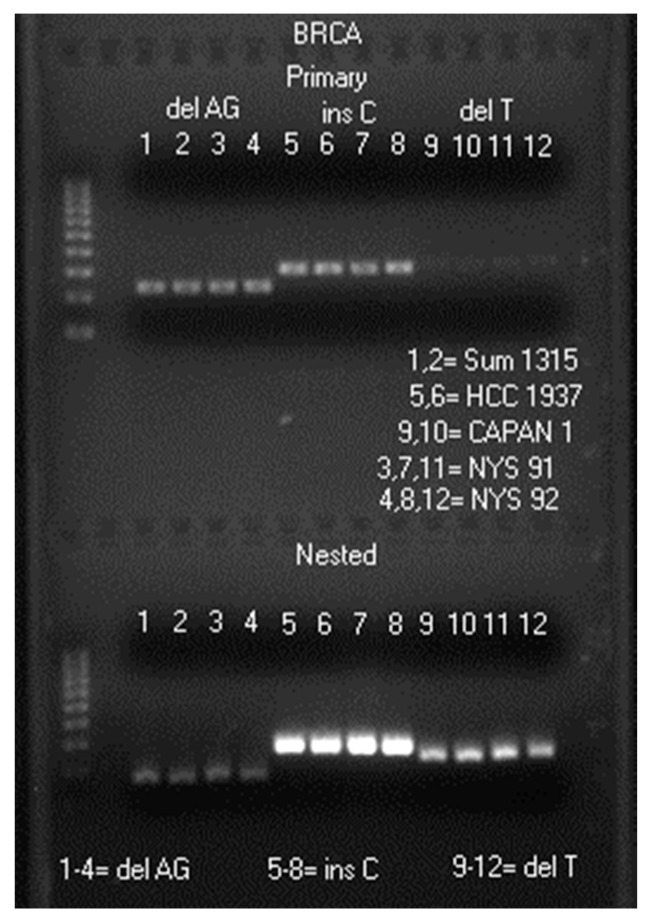
Agarose gel electrophoresis showing a 242 bp primary PCR amplicon and a 178 bp heminested PCR amplicon in lanes 1–4, a 310 bp primary PCR amplicon and a 288 bp heminested PCR amplicon in lanes 5–8, and a 322 bp primary PCR amplicon and a 247 bp heminested PCR amplicon in lanes 9–12. Molecular ruler is on the far left. delAG = amplicon for c.68_69del sequencing; insC = amplicon for c.5266dup sequencing; delT = amplicon for c.5946del sequencing. Sum1315 = cell line SUM1315M02; HCC 1937 = cell line HCC 1937, and CAPAN 1 = cell line CAPAN 1 (all fixed in Surepath™ preservatives). NYS 91 and NYS 92 = samples of human cervicovaginal cells in ThinPrep^®^ fixatives from 2015 New York State Department of Health, Clinical Laboratory Evaluation Program (CLEP). Each well was loaded with 5 μL of a mixture consisting of 5 μL heminested PCR products and 2 μL of loading fluid.

The image of a typical heminested PCR result is presented in Figure 1.

As shown in Figure 1, although the same volume (1 μL) of a cell digestate was used to initiate every PCR under identical experimental conditions, the quantities of primary PCR amplicons and nested PCR amplicons generated varied tremendously (compare the band intensities between lanes 3, 7 and 11, and those between lanes 4, 8 and 12), depending on the segment of the human genomic DNA being amplified. The target DNA amplicon in primary PCR products is usually insufficient in quantity and in purity for direct DNA sequencing without column purification or concentration. Nested PCR is needed to generate products containing enough quality PCR amplicons to be used as direct sequencing templates for HPV genotyping and for *BRCA1* c.68_69del, *BRCA1* c.5266dup and *BRCA2* c.5946del screening as further demonstrated below.

During the development phase of this testing methodology, all heminested PCR amplicons with or without a mutation were sequenced from both ends, using the forward heminested PCR primer and the reverse primer respectively as the sequencing primer to validate that the bi-directional sequencing data of the amplicons all had a 100% identity match with the consensus genomic sequences of *BRCA1* at GenBank L78833 and of *BRCA2* at GenBank Z74739. However, for routine diagnostic purpose a one-directional sequence generated with the forward nested PCR primer as the sequencing primer is adequate.

### 2.2. Routine HPV Genotyping by Partial Sanger Sequencing of the L1 Gene

**Figure 2:**
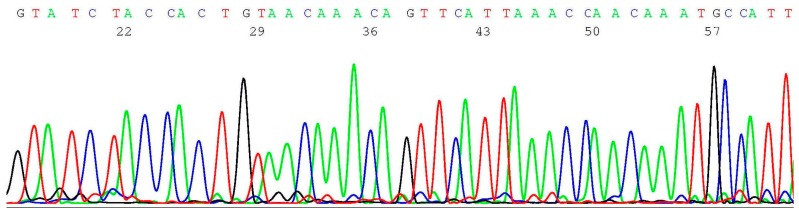
Computer-generated electropherogram showing a novel HPV-81 variant.

As previously reported, crude proteinase K digestates of cervicovaginal cells preserved in commercial ThinPrep^®^ or Surepath™ fixatives can be used for HPV detection and genotyping by Sanger sequencing [23,24,25,26,27]. To demonstrate that one proteinase K digestate can be used for HPV genotyping and *BRCA1* and *BRCA2* screening, 1 μL of the digestate of sample NYS 92 was first amplified by a pair of MY09 and MY11 degenerate primers followed by a GP6/MY11 heminested PCR primer pair, and about 0.2–0.5 μL of the nested PCR products was transferred to a 20 μL of Applied Biosystems BigDye Terminator mixture (v 1.1/Sequencing Standard) for Sanger reaction, using GP6 as the sequencing primer. This L1 gene sequencing technology not only can reliably genotype the HPV in the clinical cervicovaginal cell sample, but can also discover novel HPV variants as illustrated in this case (Figure 2).

Submission of the above 47 nucleotide bases to the GenBank for Basic Local Alignment Search Tool matching algorithm confirmed that the sequence obtained on sample NYS 92 GTATC**T**ACCACTGTAACAAACAGTTCATTAAACCAACAAATGCCATT represents a hitherto unreported HPV-81 variant with a C→T mutation (bold underlined) when aligned with the sequence in GenBank EF626590. The sequence of NYS 91 was that of HPV-61. Such novel HPV variants and less common genotypes could not have been accurately detected by the commercial hybridization-based test kits. A summary report titled “Evaluation of the New York State Human Papilloma Virus (HPV) Proficiency Test from October 2015” dated 4 December 2015 and issued by Erasmus Schneider, Ph.D., Director, Oncology Section Clinical Laboratory Evaluation Program Wadsworth Center Empire State Plaza, Albany, NY, USA states: “Not unexpectedly, there was no clear result from the genotyping of the two samples HPV091 and HPV092 that did not reach a screening consensus. Interestingly, although 95% of laboratories using Aptima^®^ classified HPV091 as screen positive, less than 10% of those that also did genotyping were able to detect either HPV 16 or 18. This is consistent with results from Roche Cobas^®^ that indicate that this sample predominantly contained HPV genotypes other than 16 or 18, in agreement with the genotypes reported from the one laboratory that used a linear array but did not find either HPV 16 or 18. Similar results were also obtained for sample HPV092, though a quarter of Roche Cobas^®^ users reported finding HPV 18” [28]. 

### 2.3. Documentation of Wild-Type Sequence without BRCA1 c.68_69del Mutation

**Figure 3:**
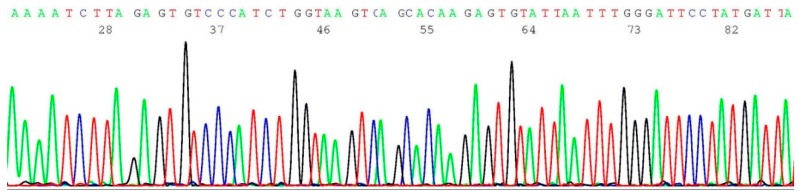
Computer-generated sequencing electropherogram of NYS 92 heminested PCR products showing no *BRCA1 c.68_69del* mutation*.* The characteristic wild-type sequence of AAAATCTTAGAGTGTCCC is clearly documented in the left end of the tracing.

About 0.2–0.5 μL of the heminested PCR products of NYS 92 (sample illustrated in lane 4 of Figure 1) was transferred by micro-glass rod to 20 μL of BigDye*^®^* Terminator mixture for sequencing reaction, using oligonucleotide 5′-TCATTGGAACAGAAAGAAATGG-3′ as the sequencing primer. A segment of the computer-generated base-calling electropherogram was excised and is presented in Figure 3.

### 2.4. Validation of BRCA1 c.68_69del Mutation by Sanger Sequencing

**Figure 4:**
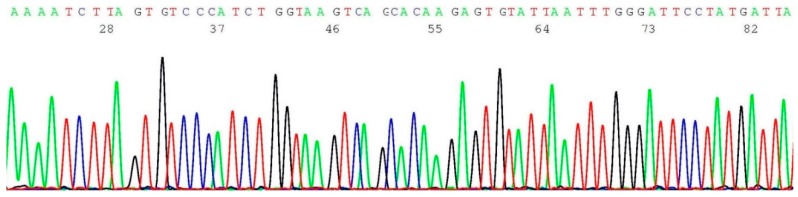
Computer-generated sequencing electropherogram of cell line SUM1315M02 heminested PCR products showing a *BRCA1 c.68_69del* mutation. The characteristic *BRCA1 c.68_69del* mutation sequence of AAAATCTTAGTGTCCC is clearly documented in the left end of the tracing (compare with Figure 3).

About 0.2–0.5 μL of the heminested PCR products of cell line SUM1315M02 (illustrated in lane 1 of Figure 1) was transferred to 20 μL of BigDye*^®^* Terminator mixture for sequencing reaction, using oligonucleotide 5′-TCATTGGAACAGAAAGAAATGG-3′ as the sequencing primer. A segment of the computer-generated base-calling electropherogram was excised and is presented in Figure 4.

### 2.5. Documentation of Wild-Type Sequence without BRCA1 c.5266dup Mutation

**Figure 5:**
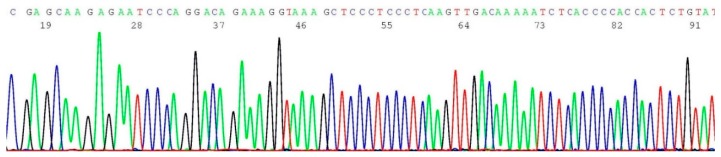
Computer-generated sequencing electropherogram of NYS 92 heminested PCR products showing no *BRCA1 c.5266dup* mutation*.* The characteristic wild-type sequence of CGAGCAAGAGAATCCCAGGACAGAAA is clearly documented in the left end of the tracing.

About 0.2–0.5 μL of the heminested PCR products of NYS 92 (sample illustrated in lane 8 of Figure 1) was transferred by micro-glass rod to 20 μL of BigDye*^®^* Terminator mixture for sequencing reaction, using oligonucleotide 5′-GAAGCTTCTCTTTCTCTTATCC-3′ as the sequencing primer. A segment of the computer-generated base-calling electropherogram was excised and is presented in Figure 5.

### 2.6. Validation of BRCA1 c.5266dup Mutation by Sanger Sequencing

**Figure 6:**
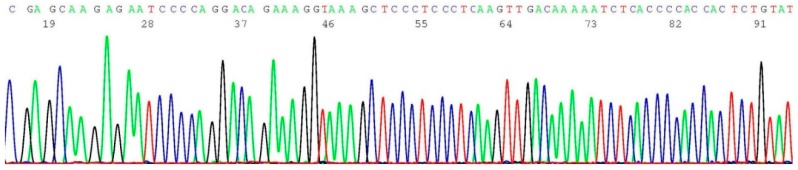
Computer-generated sequencing electropherogram of cell line HCC 1937 heminested PCR products showing a *BRCA1 c.5266dup* mutation. The characteristic *BRCA1 c.5266dup* mutation sequence of CGAGCAAGAGAATCCCCAGGACAGAAA is clearly documented in the left end of the tracing (compare with Figure 5).

About 0.2–0.5 μL of the heminested PCR products of cell line HCC 1937 (illustrated in lane 5 of Figure 1) was transferred to 20 μL of BigDye*^®^* Terminator mixture for sequencing reaction, using oligonucleotide 5′-GAAGCTTCTCTTTCTCTTATCC-3′ as the sequencing primer. A segment of the computer-generated base-calling electropherogram was excised and is presented in Figure 6.

### 2.7. Documentation of Wild-Type Sequence without BRCA2 c.5946del Mutation

**Figure 7:**
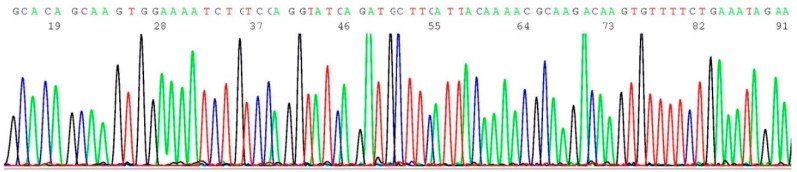
Computer-generated sequencing electropherogram of NYS 92 heminested PCR products showing no *BRCA2 c.5946del* mutation. The characteristic wild-type sequence of GCACAGCAAGTGGAAAATCT is clearly documented in the left end of the tracing.

About 0.2–0.5 μL of the heminested PCR products of NYS 92 (sample illustrated in lane 12 of Figure 1) was transferred by micro-glass rod to 20 μL of BigDye*^®^* Terminator mixture for sequencing reaction, using oligonucleotide 5′-TCACCTTGTGATGTTAGTTTGG-3′ as the sequencing primer. A segment of the computer-generated base-calling electropherogram was excised and is presented in Figure 7.

### 2.8. Validation of BRCA2 c.5946del Mutation by Sanger Sequencing

**Figure 8:**
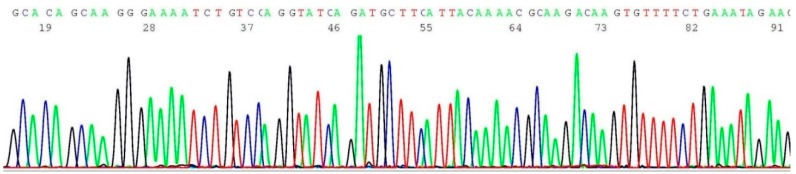
Computer-generated sequencing electropherogram of cell line CAPAN 1 heminested PCR products showing a *BRCA2 c.5946del* mutation. The characteristic *BRCA2 c.5946del* mutation sequence of GCACAGCAAGGGAAAATCT is clearly documented in the left end of the tracing (compare with Figure 7).

About 0.2–0.5 μL of the heminested PCR products of cell line CAPAN 1 (illustrated in lane 9 of Figure 1) was transferred to 20 μL of BigDye*^®^* Terminator mixture for sequencing reaction, using oligonucleotide 5′-TCACCTTGTGATGTTAGTTTGG-3′ as the sequencing primer. A segment of the computer-generated base-calling electropherogram was excised and is presented in Figure 8.

### 2.9. Challenges of Translating Science into Clinical Practice

As demonstrated above, the science of screening the three *BRCA1* and *BRCA2* founder mutations in the patient’s Pap smear cytology sample by Sanger sequencing is straightforward. Since every laboratory report would be accompanied by three DNA sequencing electropherograms as physical evidence to document the presence of a wild-type sequence or a sequence with mutation in each of these three target gene segments, interpretation of the test results is also simplified.

The PCR primers used for this method were developed for screening the three founder mutations prevalent in populations with Ashkenazi Jewish ancestry. Currently, the College of American Pathologists (CAP) and the American College of Medical Genetics (ACMG) have only regulated molecular testing for these three *BRCA1* and *BRCA2* Ashkenazi Jewish founder mutations by offering proficiency testing materials to all laboratories performing *BRCA1* and *BRCA2* mutation screening [29].

Massive next-generation sequencing technologies may generate more information on *BRCA1* and *BRCA2* mutations [7,8,9,10] and may become the platform for all commercial genetic tests in the future at a price of less than US $200 per test. However, at the present time it is recommended that all apparently positive tests be validated by standard Sanger sequencing of the patient’s DNA [30]. According to a Proficiency Testing Surveys report issued by the CAP and ACMG [29], the majority (76%) of the laboratories performing *BRCA1* and *BRCA2* founder mutations employed Sanger sequencing as their primary testing method. The majority (5/6) of laboratory errors were “false negatives”, *i.e.*, failure to detect a mutation in the survey sample. Therefore, for quality assurance the negative result for a founder mutation should be validated by a characteristic wild-type DNA sequence as illustrated in Figure 3, Figure 5 and Figure 7.

There are thousands of missense mutations in the *BRCA1* and *BRCA2* genes, most of them genetic variants of uncertain significance [6]. If these missense mutations happen to occur in one of the targeted PCR primer-binding sites of the *BRCA* gene, it may lead to failure of the PCR amplification and require newly designed PCR primers for sequencing template preparations.

One of the major concerns is that population screening for *BRCA1* and *BRCA2* mutations may overwhelm the capacity of existing genetic counselors in the United States [11]. Although board certified obstetricians and gynecologists are qualified to order genetic testing and to counsel patients prior to and after testing [31], this added workload for genetic testing may further tax the overburdened primary care clinicians [11]. The cost-effectiveness of population-based screening for these three founder mutations cannot be reliably analyzed until more data are available.

## 3. Experimental Section

### 3.1. Sources of Materials

The human cervicovaginal cell suspensions used for this study were the liquid Pap cytology specimens preserved in alcohol-based ThinPrep*^®^* (Hologic, Inc. Marlborough, MA, USA) or Surepath™ (BD Diagnostics—TriPath, Burlington, NC, USA) fixatives. Parts of these specimens had been used for HPV assays in the past 9 years as reported previously [23,24,32]. Five human cervicovaginal cell suspensions in ThinPrep*^®^* fixatives received from the New York State Department of Health Clinical Laboratory Evaluation Program (CLEP), including NYS 91 and NYS 92 presented in the Result and Discussion section were also included in this study.

The cell lines used as positive controls were the cultured cells SUM1315M02, a known carrier of *BRCA1* c.68_69del mutation [33], the cultured cells HCC1937 (ATCC*^®^* CRL-2336™), a known carrier of *BRCA1* c.5266dup mutation, and the cultured cells of Capan-1 (ATCC*^®^* HTB-79™), a known carrier of *BRCA2* c.5946del mutation. The SUM1315M02 cells were purchased from Asterand US Acquisition Corporation (Detroit, MI, USA) and the latter two from American Type Culture Collection (Manassas, VA, USA), respectively. These cultured cells were first centrifuged to remove the culture media. The cell pellet was washed in 0.85% NaCl solution, and then fixed in Surepath™ fixatives. The fixed cultured cells were processed as positive controls along with the fixed cervicovaginal cells from anonymized patients under identical experimental conditions.

### 3.2. Cell Digestion

The fixed cervicovaginal cells and cultured cells were digested according to the method developed for routine HPV detection and genotyping [23,24,25]. Briefly, an aliquot of the cell suspension containing ~5000 fixed cultured cells, or about 5% of the cell collection in a ThinPrep*^®^* or Surepath™ vial with variable cellularity, was first centrifuged for 5 min at ~16,000× *g*. The pelleted cells were washed in 1 mL reagent grade water, then in 1 mL buffer consisting of 50 mM Tris-HCl, 1 mM EDTA, 0.5% Tween 20, pH 8.1. The washed cells were re-suspended in 100 μL of 0.1 mg/mL proteinase K dissolved in the same washing buffer and digested at 45–55 °C overnight. After denaturing the proteins at 95 °C for 10 min, the digestate was centrifuged at ~16,000× *g* for 5 min. The supernatant was carefully pipetted out for PCR without further purification or stored at −20 °C until use.

### 3.3. Preparation of Heminested PCR Amplicons for DNA Sequencing

Each primary PCR mixture contained 1 μL of the sample proteinase K digestate, 1 μL of primary PCR forward primer (10 μM), 1 μL of primary PCR reverse primer (10 μM), 20 μL of LoTemp*^®^* ready-to-use master mix (Cat. No. #8802, HiFi DNA Tech, LLC, Trumbull, CT, USA) and 2 μL of deionized water to reach a total 25 μL reaction volume. For thermocycling, the temperature steps were programmed for an initial heating at 85 °C for 10 min, followed by 30 cycles at 85 °C for 30 s, 50 °C for 30 s and 65 °C for 1 min. The final extension was 65 °C for 10 min. The heminested PCR mixture contained 1 μL of heminested PCR forward primer and 1 μL of heminested PCR reverse primer in 10 μM solution, plus 20 μL of LoTemp*^®^* ready-to-use master mix and 3 μL of deionized water in a total 25 μL volume. About 0.5 μL of the primary PCR products were transferred into the correspondent heminested PCR mixture with a micro-glass rod. The thermocycling steps were identical to those used for the primary PCR.

### 3.4. DNA Sequencing

The heminested PCR products were used as the template for direct automated DNA sequencing without purification [24,25]. Briefly, 0.2–0.5 μL of the nested PCR products was carried over from the nested PCR tube with a calibrated micro-glass rod and mixed into a Sanger reaction mixture consisting of 1 μL of 10 μM sequencing primer (a forward heminested PCR primer or a reverse PCR primer), 1 μL of BigDye Terminator (v 1.1/Sequencing Standard Kit, Applied Biosystems, Foster City, CA, USA), 3.5 μL 5× buffer, and 14.5 μL of molecular grade H_2_O. Analysis of the reaction products was carried out according to the protocol supplied by the manufacturer (Applied Biosystems) in an ABI 3130 Genetic Analyzer. The DNA sequence on the computer-generated base-calling electropherogram was aligned against the standard sequences of the *Homo sapiens BRCA1* or *BRCA2* gene retrieved from the National Center for Biotechnology Information (GenBank, Bethesda, MD, USA).

### 3.5. Cross Contamination Control

To reduce the risk of PCR product cross contamination, three separate rooms with no air re-circulation, each with its own equipment and consumable supplies, were dedicated to the nucleic acid amplification and analysis. Transferring of post-PCR products was carried out in a 32” PCR workstation (AirClean Systems, Raleigh, NC, USA) in the two separate PCR rooms. Gel electrophoresis and DNA sequencing were performed in the third separate room. The pre-PCR working space was never exposed to any post-PCR materials or any items potentially contaminated by PCR amplicons.

Transferring of PCR products was always accomplished by a standardized micro-glass rod to avoid PCR product aerosol induced by micropipetting [25]. All technologists were required to pass an in-house proficiency test before being allowed to work independently.

## 4. Conclusions

We have designed three sets of heminested PCR primers to amplify three target DNA fragments in the crude proteinase K digestate of fixed cervicovaginal cells to be used as templates for direct Sanger sequencing screening of *BRCA1* c.68_69del, *BRCA1* c.5266dup and *BRCA2* c.5946del mutations. Since heminested PCR amplification is extremely effective in primer-directed target DNA enrichment [25], the number of copies of human genomic DNA available in the primary PCR is not critical for generating a mass of desired amplicon molecules in the heminested PCR products. With careful selection of the relatively inexpensive generic chemical reagents, the frontend pre-PCR sample purification and DNA quantitation can be eliminated. Since very little inhibitors are carried over from the original crude proteinase K cell digestate, the heminested PCR products can be used as the template for direct automated Sanger sequencing without post-PCR purification. With further regulatory validation, screening for *BRCA1* c.68_69del, *BRCA1* c.5266dup and *BRCA2* c.5946del mutations by Sanger sequencing can be performed at an affordable cost in conjunction with Pap smear cytology and HPV assays for evaluation of the risks of cervical cancer, ovarian cancer and breast cancer among selective patients.
